# The amplitude-amplitude cross-frequency coupling method: a step-by-step guide to quantifying physiological network interactions

**DOI:** 10.3389/fnetp.2026.1784539

**Published:** 2026-04-10

**Authors:** Sergi Garcia-Retortillo, Óscar Abenza, Yaopeng J. X. Ma, Plamen Ch. Ivanov

**Affiliations:** 1 College of Nursing, University of Central Florida, Orlando, FL, United States; 2 Faculty of Medicine and Health Sciences, University of Barcelona, Barcelona, Spain; 3 Keck Laboratory for Network Physiology, Department of Physics, Boston University, Boston, MA, United States; 4 Department of Neurosurgery, Boston University Chobanian and Avedisian School of Medicine, Boston, MA, United States; 5 Institute of Biophysics and Biomedical Engineering, Bulgarian Academy of Sciences, Sofia, Bulgaria

**Keywords:** complex systems, dynamic networks, electrocardiography, electromyography, electrophysiology, exercise, network physiology

## Abstract

The human organism operates as an integrated network in which multiple physiological systems dynamically coordinate across spatial and temporal scales. Quantifying these interactions requires analytical frameworks that move beyond single-system measures and capture multisystem coordination. Here, we present a detailed, step-by-step description of the Amplitude-Amplitude Cross-Frequency Coupling (ACFC) method, a network-based approach designed to quantify coordination among skeletomuscular, cardiovascular, and respiratory systems using simultaneous electrophysiological recordings. ACFC evaluates how the amplitudes of oscillatory components across specific frequency bands co-vary over time, producing three network-based markers: inter-muscular, cardio-muscular, and respiratory-muscular coupling. The method combines spectral decomposition, cross-correlation analyses, and network dynamics to characterize global network organization and coupling strength for distinct physiological states, and the temporal variability in systems coordination and network interactions at short timescales. Beyond quantifying average coupling and network link strength over extended period of time associated with a given physiological state, ACFC enables probing the temporal coordination of physiological rhythms embedded in systems dynamics, as well as the variability and evolution of their network interactions across timescales and states in response to internal and external demands. Using a bodyweight squat protocol as an illustrative example, we outline all analytical steps, parameter choices, and practical considerations required to implement the ACFC method to quantify physiological systems coupling and network interactions. This Methods article provides a reproducible guide for applying ACFC analyses and is intended to facilitate the adoption, adaptation, and extension of network-based approaches to study multisystem coordination in exercise, aging, and broader physiological or clinical contexts in Network Physiology.

## 1 Introduction

The human organism functions as an integrated network in which multiple physiological systems—such as the skeletomuscular, cardiovascular, respiratory—continuously coordinate and synchronize their dynamics across timescales and organizational levels (Bartsch et al., 2015; Liu K. K. L. et al., 2015; Ivanov et al., 2017; Rizzo et al., 2020). Disruptions in physiological network coordination can lead to dysfunction in individual systems or to a breakdown in organism-level regulation (Rizzo et al., 2023). However, most of the existing physiological research has primarily focused on individual physiological systems, their structural and dynamic complexity, thus, limiting the ability to capture coordinated dynamics and network interactions among systems. To address this gap, the multidisciplinary field of Network Physiology was established (Bashan et al., 2012; Ivanov and Bartsch, 2014; Ivanov, 2021), providing a conceptual and quantitative framework to investigate how diverse physiological systems coordinate as a dynamic network to generate distinct physiological states in health and disease (Bartsch et al., 2015; Liu K. et al., 2015; Ivanov et al., 2016; Lin et al., 2016; Ivanov et al., 2021b). Within the Network Physiology framework, physiological function is understood not only in terms of system-specific activity, but also through dynamic coordination patterns and global network interactions that characterize distinct physiological states and functions.

Several analytical tools derived from statistical physics and nonlinear dynamics have been developed to quantify and infer nonlinear interactions among systems, including time-delay stability (Bashan et al., 2012; Bartsch et al., 2014; Liu K. et al., 2015; Marzbanrad et al., 2020; Ivanov et al., 2021a), phase synchronization (Schäfer et al., 1998; Chen et al., 2006; Bartsch et al., 2012), coherence (Chorlian et al., 2009; McCraty et al., 2009; Bian et al., 2014), complex wavelets (La Rocca et al., 2021), mutual information (Faes et al., 2014; Valente et al., 2018; Antonacci et al., 2020), transfer entropy (Dumont et al., 2004; Faes et al., 2015; Valenza et al., 2016; Lucchini et al., 2020), or Granger causality (Katoh et al., 2014). Motivated by earlier empirical works on cortical rhythm networks and cortico-muscular networks (Bartsch et al., 2015; Liu K. et al., 2015; Lin et al., 2020; Rizzo et al., 2020; 2023; Chen et al., 2022), the Amplitude-Amplitude Cross-Frequency Coupling (ACFC) method has been introduced in the context of Network Physiology of Exercise (Balagué et al., 2020; Balagué et al., 2022a; Balagué et al., 2022b) to evaluate how the amplitudes of oscillatory components in different physiological signals co-vary during exercise (Garcia-Retortillo and Ivanov, 2022; 2025; Garcia-Retortillo et al., 2023; Garcia-Retortillo et al., 2025a; Abenza et al., 2025).

**FIGURE 1:**
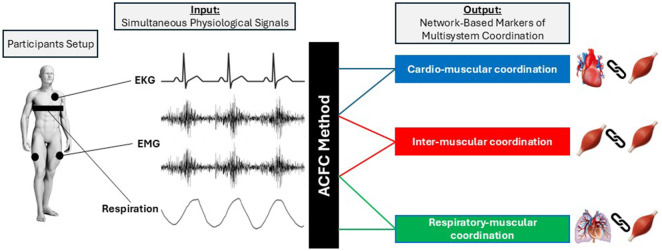
Schematic representation of the participants setup, input signals and output network markers derived from ACFC analyses.

**FIGURE 2:**
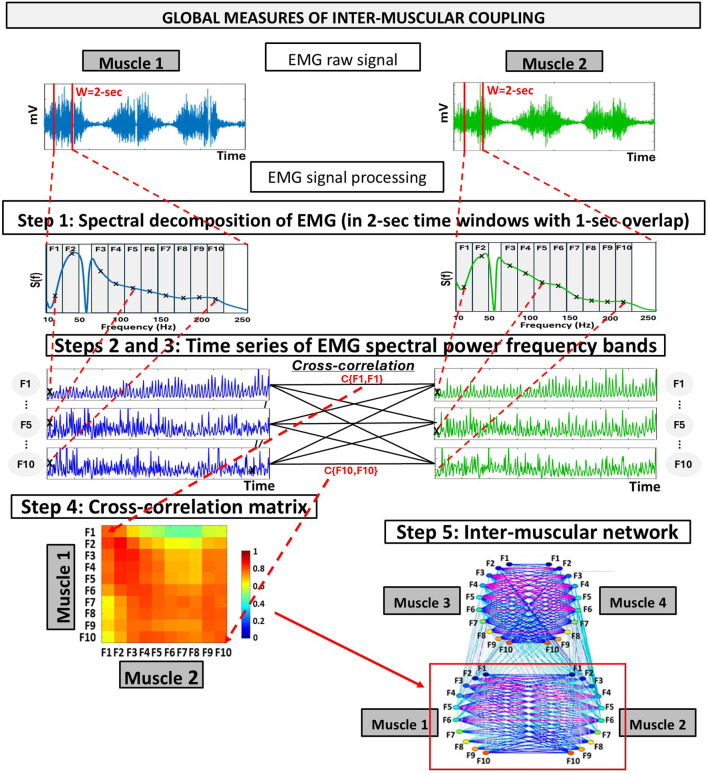
Schematic diagram representing the analyses for the global measures of inter-muscular coupling using the Amplitude-Amplitude Cross-Frequency Coupling (ACFC). Step 1: Raw EMG signals are obtained from two different muscles over the same period. Step 2: Spectral decomposition is performed on each 2-s time window (with 1-s step), using 0.5 Hz bins within the 10–250 Hz range. To capture the specific contribution of different frequency bands, the resulting spectrum is divided into ten frequency bands of equal width (19.5 Hz), and the power within each frequency band is summed to obtain a single value per frequency band and window. Step 3: This process generates a time series of spectral power for each frequency band, sampled at 1-s intervals (i.e., 1-s resolution). Bivariate equal-time Pearson’s cross-correlation are then computed at zero lag (no delay) between all combinations of frequency band time series from the two muscles. Step 4: This results in a 10 × 10 cross-correlation matrix (*C*_*m,k*_) representing the coupling among frequency bands between the two muscles. Step 5: Inter-muscular networks are constructed from the cross-correlation matrices of all muscle pairs (sub-networks). In these networks, nodes represent EMG frequency bands, and link strength reflects the degree of coupling between frequency bands across muscles. Note that this schematic illustrates the steps for inter-muscular coupling. While the same procedures apply to cardio-muscular and respiratory-muscular coupling, some particularities should be considered (see Sections 3.4, 3.5).

**FIGURE 3:**
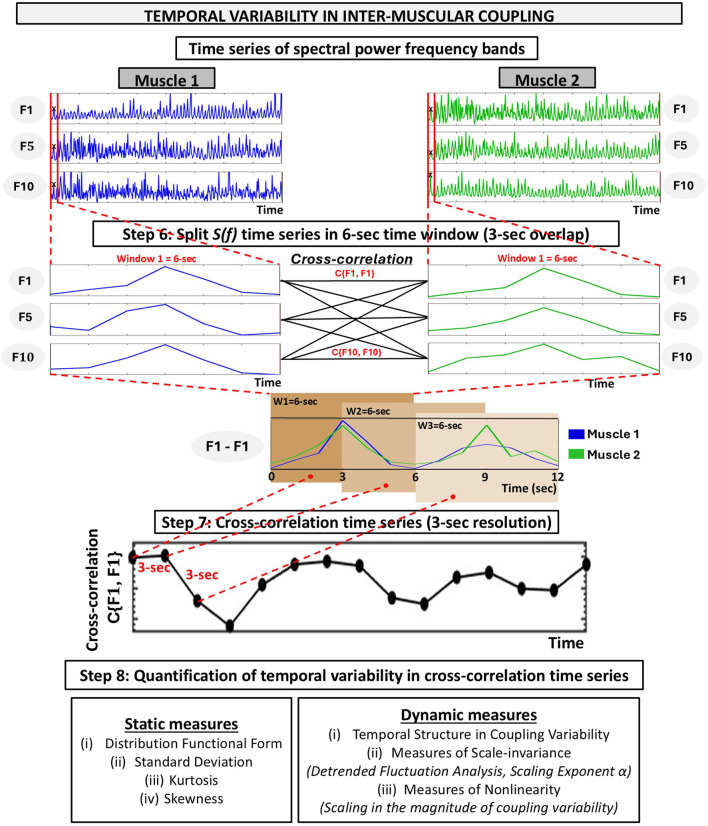
Schematic diagram representing the analyses for the temporal variability in inter-muscular coupling using the amplitude-amplitude cross-frequency coupling (ACFC). Previous steps: Spectral decomposition of EMG signals and computation of time series of EMG spectral power frequency bands, as described in Figure 2 (Steps 1-5). Step 6: Each time series is divided into windows of 6-s (with 3-s step), resulting in *NL* segments of length *L* = 3-s (*NL* = [*N/L*]), where *N* is the length of the time series and *L* the step size. For each 6-s segment, bivariate equal-time Pearson’s cross-correlation are computed at zero lag (no delay) across all combinations of frequency band time series pairs between the two muscles. Step 7: This results in 10 × 10 cross-correlation values $C_{\tau =0}\left(t_{i}\right)\left\{F_{m},F_{k}\right\}$ for each segment. This generates a cross-correlation time series for each link (i.e., frequency band pair combination) between the two muscles, representing the temporal variability in inter-muscular coupling among frequency bands between the two muscles. Step 8: Quantification of temporal variability in cross-correlation time series. The resulting cross-correlation time series for each frequency-band combination (i.e., link) are used to quantify both the magnitude (static measures) and the temporal structure (dynamic measures; Ashkenazy et al., 2001; 2003; Kantelhardt et al., 2002) of inter-muscular coupling variability. Note that this is an example of the different steps for cross-correlation time series using inter-muscular coupling. While the same procedures apply to cardio-muscular and respiratory-muscular coupling, some particularities should be considered (see Sections 3.4 and 3.5).

The ACFC uses simultaneous physiological signals, such as (but not limited to) electromyography (EMG), electrocardiography (EKG), and respiration, as inputs to quantify multisystem coordination across skeletal muscles, the cardiac, and respiratory system. Specifically, ACFC measures pairwise coupling between muscle–muscle, heart–muscle, and respiration–muscle. The final outcome of the analyses are three network-based markers: inter-muscular, cardio-muscular, and respiratory-muscular coupling (Figure 1). The method is implemented in two phases: Phase 1 (Figure 2) provides global measures of coupling within a defined time segment (e.g., Rest, Exercise), while Phase 2 (Figure 3) captures the micro-scale temporal variability of coupling, quantifying how this coupling fluctuates and reorganizes instantaneously over time. Together, Phase 1 and Phase 2 of the ACFC method offer a comprehensive characterization of both the magnitude and temporal structure of multisystem coordination.

Whereas traditional analyses evaluate physiological systems independently, ACFC-derived markers quantify how multiple muscles, the heart, and respiration coordinate as an integrated network. In our recent work, these markers have been applied to investigate multisystem coordination in response to fatigue, aging, and comparing between males and females across different movements and exercise protocols (Garcia-Retortillo and Ivanov, 2022; 2025; Garcia-Retortillo et al., 2023; 2025b; 2025a; Abenza et al., 2025). These applications highlight ACFC as a versatile tool for probing multisystem coordination during rest, exercise, and broader physiological or clinical conditions.

Despite its potential, implementing ACFC requires a sequence of specific signal-processing and analytical steps that are not currently available as a standard toolbox and must be scripted by the investigator. The goal of this Methods article is therefore to provide a complete, step-by-step description of the ACFC framework—from data acquisition and spectral decomposition to the construction of inter-muscular, cardio-muscular, and respiratory-muscular networks. By detailing the required materials, preprocessing steps, parameter choices, and analytical procedures, this manuscript aims to enable researchers to reproduce ACFC analyses, adapt them to different experimental protocols and populations, and extend the method to new questions in physiology and related biomedical fields.

## 2 Materials and equipment

### 2.1 Hardware and consumables

The ACFC method requires physiological signals that are recorded simultaneously and continuously at high sampling frequencies and with stable temporal resolution, as the method relies on capturing coordinated rapid fluctuations in the amplitude of oscillatory components embedded in the signals. For this reason, electrophysiological signals such as EMG, EKG, and respiratory waveform recordings are particularly well suited for ACFC analyses. In contrast, physiological variables derived from slower physiological monitoring systems—such as gas exchange measurements obtained from metabolic carts (e.g., oxygen uptake, carbon dioxide production, or ventilation) or other commonly utilized measures for physiological assessment—are typically computed over longer averaging intervals and therefore lack the temporal resolution required to construct high-frequency time series suitable for ACFC analysis.

For EMG and EKG acquisition, bipolar surface electrodes were used (Meditrace Foam EG200, Danlee Medical Products Inc., Syracuse, United States). These electrodes were selected due to their stable adhesion, low impedance, and suitability for dynamic movements; however, any high-quality Ag/AgCl bipolar electrodes with comparable characteristics are appropriate for ACFC data collection. Standard skin preparation materials are required, including alcohol wipes for reducing impedance at electrode sites and conductive gel to optimize EKG signal quality.

Together, these components provide all hardware and consumables needed for simultaneous acquisition of muscle, cardiac, and respiratory electrophysiological signals to implement the ACFC method.

### 2.2 Software

In this work, and in our previously published studies, all signal processing and ACFC computations were performed using MATLAB (MathWorks, Natick, MA, United States). MATLAB was selected due to its robust capabilities for handling high-frequency electrophysiological data, advanced spectral analysis functions, and flexible scripting environment. However, any scientific computing software capable of processing high-sampling-rate time series is fully suitable for implementing the ACFC method.

Because the ACFC framework is not currently available as a pre-packaged toolbox in any platform, all analyses must be implemented through custom scripts. As long as investigators follow the procedures, equations, and processing steps detailed in this manuscript, the method can be reproduced in any environment that supports: segmentation of high-frequency time series, spectral decomposition (e.g., Welch’s method), correlation analysis, statistical and nonlinear dynamics analyses.

## 3 Methods

Here we present the ACFC method using a specific experimental example that combines a particular exercise movement (bodyweight squat), a defined protocol including an exercise bout performed until exhaustion to assess fatigue, and recordings from a set of leg and back muscles, heart rate and respiratory rate. These choices are made solely for illustrative purposes to facilitate the step-by-step description of the analytical procedure. Importantly, the ACFC method is not restricted to this specific protocol and can be applied to other experimental designs involving different movements, muscles, and simultaneous physiological signals, depending on the objectives of the study. The figures presented in the Expected Results section are illustrative examples derived from previous studies conducted by our group under protocols approved by the local Institutional Review Board (WFU IRB00024843). No new experimental data are reported in this Methods manuscript.

Given the nature of ACFC, it is recommended to use a cyclic movement with a predefined tempo and a consistent range of motion throughout the entire protocol.

After presenting the experimental protocol and participants set up, we outline the steps for each of the network-based markers that are the outcomes of the ACFC analyses: inter-muscular (see Section 3.4), cardio-muscular (see Section 3.5), and respiratory-muscular coupling (see Section 3.6). While the overall procedures are similar across the three markers, there are some subtle differences that must be considered.

### 3.1 Study design and experimental protocol

The squat test was selected as a representative protocol because trunk–leg coordination is essential for many functional tasks, making it a suitable exercise to quantify multisystem coordination. The squat has been proposed as an accessible and reliable tool for assessing multisystem coordination (Bianco et al., 2015).

To control movement tempo and ensure consistency across participants, a metronome (MetroTimer version 3.3.2, ONYX Apps) was used to regulate a 3:3 rhythm, with 3-s for the downward phase and 3-s for the upward phase, making each squat last for a total of 6s. In our protocol, squat movements during each exercise segment continued until participants were unable to perform the next squat or maintain the required 3:3 squat tempo.

The squat movement was executed according to the following instructions: participants were instructed to position their feet slightly wider than shoulder-width apart, extend their arms straight forward, and initiate the descent inhaling while unlocking the hips and gently pushing them backwards. As the knees began to flex, they continued lowering themselves until reaching a guiding rope that ensured a consistent range of motion. The guiding rope was set at a height corresponding to the participants’ thighs when parallel to the ground at the lowest squat position. From this point, participants ascended to a completely extended position before initiating the next repetition. Throughout the movement, they were instructed to maintain an upright chest, keep their weight distributed over their heels, and avoid their knees from moving inward (Bianco et al., 2015).

### 3.2 Sensors set up (EMG, EKG, respiration)

Participants were asked to wear appropriate clothing that would not obstruct electrode placement sites. Before the mounting of the electrodes, participants’ skin was shaved and cleaned using alcohol and left to dry for 60s to reduce the myoelectrical impedance (Hermens et al., 2000). Three different output signals were collected simultaneously during the entire experimental protocol. (1) Surface electromyography (EMG) signals from left and right vastus lateralis (Leg-Left and Leg-Right muscles), and left and right erector spinae longissimus (Back-Left and Back-Right). The exact location of the surface electrodes (Meditrace Foam EG200, Danlee Medical Products Inc., Syracuse, United States) placement on each muscle was carried out according to the SENIAM recommendations (Hermens et al., 2000). These muscles have been selected because they represent primary movers of the lower limbs (vastus lateralis) and key postural stabilizers of the trunk (erector spinae), both of which are essential for locomotion, balance, and functional mobility (Khaiyat and Norris, 2018). (2) Electrocardiography (EKG) signal using a standard 3-lead EKG set-up to obtain the recording for lead II: right shoulder (−), left shoulder (ground) and lower left abdomen (+). (3) Respiration was measured using a respiration belt equipped with a precision transducer to detect changes in thoracic circumference. The belt was securely positioned around the upper thorax, approximately at the level of the axillae. After the electrodes and belt are secured to minimize movement artefacts, a signal quality check was performed to ensure signal validity. EMG and EKG electrodes, as well as the respiratory belt, were remain attached to the participant throughout the entire experimental protocol.

### 3.3 Basic signal processing

Before applying the ACFC method, raw signals should undergo basic preprocessing. Because ACFC relies on frequency-specific analyses, the optimal preprocessing parameters—particularly the band-pass filter settings—depend on the EMG frequency bands selected by the researcher (see Steps 1 and 2 in Section 3.4.1 of the Methods). For example, investigators interested only in low-frequency EMG components may use narrower band-pass ranges, whereas studies focusing on higher frequencies activity require broader upper cutoffs. Thus, while recommended defaults are provided here, filtering parameters should be adapted to the goals and spectral range of each study.

In our work, signal preprocessing included band-pass filtering between 0.5–150 for EKG and 5–250 for EMG. Additionally, a notch filter with a width of 1 Hz at 60 Hz was applied to both EKG and EMG signals to eliminate power line interference from the grid. The notch filter should be adjusted to local electrical grid frequency (e.g., 50 Hz in most European countries).

### 3.4 Inter-muscular coupling and network interactions

#### 3.4.1 Global measures of inter-muscular coupling

The steps of this section are summarized in Figure 2.

Step 1: Spectral decomposition of EMG signals

The EMG signals recorded from the left and right vastus lateralis (Leg-Left and Leg-Right muscles) and the left and right erector spinae longissimus (Back-Left and Back-Right) were segmented into 2-s time windows with a 1-s step. Within each time window, the spectral power $S\left(f,t_{i}\right)$ of the EMG signals was extracted using the ‘pwelch’ function in MATLAB, which is based on the discrete Fourier transform and Welch’s overlapped segment averaging estimator. Given that each 2-s window was analyzed as a single segment, this procedure is equivalent to a short-time Fourier transform applied with the same window length and step. The spectral power $S\left(f,t_{i}\right)$ was computed in bins of 0.5 Hz within the range of [10–250 Hz], resulting in N = 480 spectral power data points per 2-s window.

To investigate the specific contributions of different frequency components to the EMG spectral power, the extracted spectra were divided into ten frequency bands (*F*_*m*_), each with an equal width of 19.5 Hz. This frequency segmentation was based on previous research analyzing the spectral power profiles of the vastus lateralis and erector spinae muscles during squat exercises and identified physiologically relevant information distributed within the 10–230 Hz range (Garcia-Retortillo et al., 2020), where different frequency bandwidths—broad (50–100 Hz), intermediate (20 Hz), and narrow (5 Hz)—were evaluated. Both the intermediate and narrow bands preserved physiologically relevant information in the signal, whereas the broader bandwidth showed lower physiological sensitivity. Because the intermediate and narrow bands provided similar physiological detail, a 20-Hz bandwidth was proposed as most efficient option for characterizing the contribution of distinct frequency components within the EMG spectral distribution during exercise.

Accordingly, we selected ten frequency bands of equal width (19.5 Hz) to divide the 10–230 Hz range. The 50–65 Hz interval was excluded from the analysis because it was affected by the notch filter applied at 60 Hz to remove power-line interference. As a result, the analyzed bands are not fully contiguous. Importantly, this omission means that transitions between neighbouring bands around the excluded region should not be interpreted as continuous spectral changes, since part of the spectrum was intentionally removed due to preprocessing. Therefore, coupling patterns involving bands adjacent to this interval should be interpreted with this discontinuity in mind.

Step 2: EMG frequency bands time series

The spectral power for each frequency band *F*_*m*_ (where m=1,…,10 ) at a given time window centered at $t_{i}$ was computed as the sum of the power across all discrete frequency bins $f_{j}$ within that corresponding band:

$$P\left(F_{m},t_{i}\right)=\sum_{f_{j}\in F_{m}}S\left(f_{j},t_{i}\right)$$

where the summation includes all 39 discrete frequency bins ( $f_{j}$ ) contained within the specific frequency band $F_{m}$ . This procedure resulted in ten EMG band power time series with 1-s resolution for each muscle during the protocol (e.g., Rest, Exercise).

To examine the contribution from different frequency bands $F_{m}$ to the EMG spectral power within each 2-s window, we defined 10 frequency bands with equal width of 19.5 Hz: F1 = [10–29.5 Hz], F2 = [30–49.5 Hz], F3 = [65–84.5 Hz], F4 = [85–104.5 Hz], F5 = [105–124.5 Hz], F6 = [125–144.5 Hz], F7 = [145–164.5 Hz], F8 = [165–184.5 Hz], F9 = [185–204.5] and F10 = [205–224.5 Hz]. Notably, the relationship between EMG spectral power frequency bands and muscle fiber types is not straightforward, as spectral power is influenced by various factors such as motor unit synchronization, tissue filtering effects, and electrode placement (Farina, 2008; Von Tscharner and Nigg, 2008; Del Vecchio et al., 2017). Nevertheless, lower-frequency components (<30–40 Hz; F1–F2) are consistent with motor units exhibiting lower muscle fiber conduction velocities and longer-duration motor unit action potentials (MUAPs), features typically associated with low-threshold motor units, which are often composed predominantly of slow-twitch fibers. In contrast, recruitment of higher-threshold motor units—generally characterized by larger axon diameters, higher conduction velocities, and shorter-duration MUAPs—broadens the EMG spectrum toward higher frequencies (Grimby et al., 1979; 1981; Wakeling et al., 2001; Dreibati et al., 2010; Rosenblum et al., 2022). Higher frequencies (above∼100 Hz) primarily reflect MUAP waveform characteristics rather than motor unit discharge rate *per se*. MUAPs consist of short-duration electrical events (approximately 5–10 ms) that contribute to the high-frequency content of the EMG signal due to: the steep rising and falling phases of MUAP waveforms (shape), the temporal summation/overlap of multiple MUAPs, and muscle fiber conduction properties (Merletti and Farina, 2016). Fast-twitch muscle fibers, which generate shorter-duration action potentials, may contribute more significantly to this higher-frequency component, extending the EMG spectral content into the 100–230 Hz range. Importantly, the defined frequency bands should not be interpreted as exclusive markers of specific fiber types, but rather as reflecting shifts in the relative contribution of motor units with differing electrophysiological properties.

The resulting time series were normalized to have zero mean (*μ* = 0) and unit standard deviation (SD; *σ* = 1). This normalization removes differences in signal magnitude across muscles, frequency bands, and physiological systems, allowing the cross-correlation analysis to capture relative fluctuations in signal dynamics rather than absolute amplitude differences.

Importantly, in the ACFC method, two parameters critically shape the spectral power time series derived from EMG: (i) the window step, and (ii) the window size. Each parameter has a distinct functional role and should be selected according to the temporal characteristics of the experimental protocol. The window step determines the temporal resolution of the EMG frequency-band time series, as it defines how frequently new spectral estimates are produced. For example, using 2-s windows with a 1-s step yields a temporal resolution of 1-s (i.e., one power value every sec). This resolution must align with the timescale of the task being studied. In our squat protocol, participants performed one squat approximately every 6-s (∼0.15 Hz). According to the Nyquist criterion, resolving this movement requires at least ∼0.3 Hz temporal resolution; therefore, a 1-s resolution was sufficient. However, protocols with faster dynamics require higher resolution. For instance, cycling at 70 revolutions per minute (RPM) (∼1.15 Hz) requires an EMG frequency-band time-series resolution higher than 2.3 Hz. Thus, the choice of window step is protocol-dependent and should match the timescale of the physiological or behavioral events under study.

The window size determines the frequency range that can be reliably analyzed. Independent of the step, the window size constrains which frequencies can be meaningfully resolved in the spectral analysis. High-frequency signals such as EMG can be characterized accurately with relatively short windows (1–2 s, depending on the selected raw sampling frequency), since skeletal muscles exhibit spectral energy up to approximately 250 Hz. According to the Nyquist criterion, the analysis window should contain a sufficient number of data points to reliably resolve these frequencies (at least 500 samples).

Another important consideration arises when EMG is coupled with a different type of physiological signal. If the second signal operates at much lower frequencies, such as respiration, the window size must be increased to capture complete physiological cycles and produce valid ACFC estimates. In general, the window size should reflect the dominant frequency of the slowest signal involved in the coupling analysis. This consideration is particularly relevant for respiratory–muscular coordination and is discussed in Section 3.6.

Step 3: Cross-correlations between time series of EMG frequency bands

To quantify coupling among EMG frequency bands across muscles, we performed cross-correlations between all pairs of muscles (LegL-LegR, LegL-BackL, LegL-BackR, LegR- BackL, LegR-BackR, and BackL-BackR).

For each muscle pair, we computed the bivariate equal-time Pearson’s cross-correlation between the time series representing EMG spectral power in the frequency bands *F*_*m*_ from the first muscle (*F*_*m*_ where *m* = 1,…,10) and the frequency bands *F*_*k*_ from the second muscle in the pair (*F*_*k*_ where *k* = 1, … ,10). This resulted to 10 × 10 = 100 cross-correlation values *C*_*m*_ *,*_*k*_ for each pair of muscles. The coefficient *C*_*m*_ *,*_*k*_ quantifies the degree of cross-correlation between the frequency band *F*_*m*_ of the first muscle and *F*_*k*_ of the second muscle, with values ranging from *C*_*m*_ *,*_*k*_ = −1 (fully anti-correlated, i.e., anti-synchronously modulated spectral power in the frequency bands $F_{k}$ and $F_{m}$ ) to *C*_*m*_ *,*_*k*_ = 1 (fully positively correlated, i.e., synchronously modulated spectral power in the frequency bands $F_{k}$ and $F_{m}$ ), while *C*_*m*_ *,*_*k*_ = 0 indicates the absence of linear correlation.

In this specific example, we defined zero-lag assumption (τ = 0) because, in the body-weight squat analysis, τ = 0 yielded higher cross-correlation coefficients than the other lag comparisons tested (τ = 1, τ = 2 …). However, this choice should not be generalized to all experimental procedures, as non-zero lags may be expected in situations involving delayed physiological responses or different tasks. Accordingly, we recommend using τ = 0 as a default, while also evaluating other lag values for the specific task under study before selecting the most appropriate lag. Based on this, we defined the cross-correlation values as a measure of relative timing, considering them a hallmark of motor coordination (Kelso and Jeka, 1992).

Step 4: Inter-muscular cross-correlation matrices

The group-averaged cross-correlation matrices illustrate the pairwise correlation between the ten frequency bands *F*_*m*_ of one muscle and the corresponding bands derived from another muscle (i.e., 6 distinct muscle pairs) during the test. More precisely, each matrix element represents the group-averaged cross-correlation calculated as the average value of the cross-correlation coefficients across all participants in the group. Consequently, each matrix comprises 100 elements (cross-correlation coefficients) that represent the interactions for each pair of frequency bands in the muscle pairs. The dimensionality of these cross-correlation matrices depends on the number of frequency bands included in the analysis (see Section 3.4.1). Measures of central tendency and dispersion can then be selected by the investigator according to the structure and characteristics of the data.

Step 5: Network maps of inter-muscular interactions

To visualize the interactions between all muscles and their hierarchical organization within the network, we constructed a multiplex network of sub-networks (i.e., muscle pairs). These network maps were generated by mapping the results of the cross-correlation analyses (see Section 3.4.1) into different group-averaged networks. A graphical approach was employed to identify the universal patterns in the inter-muscular network, and to characterize its hierarchical organization. Each network was represented as a semicircle, with color-coded nodes indicating distinct frequency bands *F*_*k*_. Network links corresponded to the cross-correlation values *C*_*m*_ *,*_*k*_ obtained from the cross-correlation analysis and represented the coupling strength between frequency bands of two different muscles within the sub-network (i.e., muscle pair). The strength of these links was visually encoded using variations in line color and width, where distinct colors were used to illustrate network reorganization. The link strength classification was established according to the obtained results, to facilitate the visualization. Example of a possible classification: (1) weak links (0.20 < *C*_*m*_ *,*_*k*_ < 0.35; very thin grey lines), (2) intermediate links (0.35 < *C*_*m*_ *,*_*k*_ < 0.5; thin green lines), (3) strong links (0.5 < *C*_*m*_ *,*_*k*_ < 0.65; thick dark blue lines), and (4) very strong links (*C*_*m*_ *,*_*k*_ > 0.65; very thick magenta lines).

To further assess the physiological relevance of the inter-muscular networks derived from the previous analysis, Fourier-based surrogate data analyses should be applied to the EMG signals, as described in previous studies (Garcia-Retortillo and Ivanov, 2022; 2025; Garcia-Retortillo et al., 2023; Abenza et al., 2025).

##### 3.4.1.1 Additional control analyses for global measures of inter-muscular coupling

To assess the physiological relevance of the inter-muscular interaction networks derived from the previous steps, we conducted a Fourier phase randomization surrogate test (Theiler et al., 1992). In this procedure, surrogate EMG signals are generated by preserving the amplitude distribution and power spectrum of the original signals across frequency bands (*F*_*m*_), while randomizing the Fourier phases. As a result, the surrogate signals retain the global spectral content and the relative distribution of power across frequency bands, but their fine temporal organization and phase-dependent structure related to nonlinear characteristics are disrupted. This allows to test whether the observed inter-muscular correlations depend on preserved temporal organization rather than on shared marginal spectral properties alone. After generating the surrogate EMG signals, we recomputed EMG spectral power time series and repeated the cross-correlation analysis following the same ACFC method steps used for the original data. Markedly reduced and clustered close to zero cross-correlation values after the Fourier phase randomization test compared to the original dataset, would indicate that cross-correlations (coupling) depend on preserved temporal organization rather than on shared marginal spectral properties.

Further, to assess the statistical significance of network links strength and the physiological relevance of the observed hierarchical network structure and its reorganization with fatigue, we added a further step in the surrogate procedure. Specifically, for each network link, surrogate values were generated using Fourier phase randomized signals from all possible pairs of randomly selected participants, thus, allowing to define a threshold for the physiological significance of links strength. For example, assuming 35 participants, all unique participant pairs were considered, yielding 595 participant combinations for each link. For each muscle pair, the corresponding sub-network included 100 links (10 × 10 links between the ten frequency bands $F_{m}$ of each muscle). Considering the six sub-networks representing all muscle pairs, this resulted in a surrogate distribution of 357,000 links strength values (i.e., 6 muscle pairs × 100 links × 595 participant combinations = 357,000). From this distribution, the surrogate mean (μ_surr) and standard deviation (σ_surr) were computed. The 95% confidence threshold for link strength was then defined as Th = μ_surr + 2σ_surr (Th = 0.11, Figure 5) (Garcia-Retortillo and Ivanov, 2022).

Finally, in exercise protocols that produce a slow monotonic incremental trend in EMG spectral power, it is important to evaluate whether such trends may artificially influence cross-correlation estimates (Podobnik et al., 2007). For this reason, we recommend performing an additional analysis in which the spectral power time series are detrended prior to computing cross-correlations. This situation may arise, for example, in incremental exercise tests performed on a cycle ergometer, where workload (watts) is gradually increased every 1–2 min until exhaustion—a protocol commonly used as a gold-standard test for assessing cardiorespiratory fitness.

#### 3.4.2 Temporal variability within states and evolution across states in inter-muscular coupling

To assess the temporal variability of inter-muscular coupling between EMG frequency bands across muscles that occur as a result of synchronous modulation of their amplitudes at short timescales of a few seconds, we followed the steps below. The steps of this section are summarized in Figure 3.

Step 6: Splitting EMG frequency bands time series into time windows

We divided the EMG frequency bands time series obtained in Step 2 into *NL* segments of 6-s of length, with a 3-s step between consecutive segments (*NL* = (*N*/*L*)), where *N* is the length of the time series and L denotes the time step between consecutive segments (i.e., 3-s). Given that each squat movement cycle lasts 6-s (3-s down and 3-s up), this segmentation ensured that each window captured the entire movement cycle. However, the window length should be adjusted according to the specific exercise. Importantly, it should be noted that time windows containing a limited number of data points may yield more variable or noisy correlation coefficient estimates, and therefore the window length should be selected carefully according to the temporal characteristics of the experimental protocol.

Importantly, in this step, the EMG frequency band time series were normalized separately within each 6-s segment to have zero mean (*μ* = 0) and unit SD (*σ* = 1). The reason for applying normalization within each short segment is that, if the entire time series were normalized using zero mean (*μ = 0*) and unit standard deviation (*σ = 1*), the relative magnitude of individual short segments could become distorted. Normalization of each 6-s segment aims to preserve the local fluctuation pattern within that window and to account for the short-timescale co-variation between signals when calculating the cross-correlation coefficient *C*_*m*_ *,*_*k*_ values.

Step 7: Inter-muscular cross-correlation time series

For each muscle pair (LegL-LegR, LegL-BackL, LegL-BackR, LegR- BackL, and LegR-BackR, BackL-BackR), we calculated the bivariate equal-time Pearson’s cross-correlation between the spectral power time series of all 10 EMG frequency bands (*F*_*m*_, where *m* = 1, … ,10) within each 6-s segment. Let $\hat{P^{\left(1\right)}}\left(F_{m},t_{i,j}\right)$ and $\hat{P^{\left(2\right)}}\left(F_{k},t_{i,j}\right)$ denote the locally normalized spectral power values (as described in Step 6, with zero mean and unit variance) for frequency band $F_{m}$ of the first muscle and $F_{k}$ of the second muscle. For a 6-s segment centered at time $t_{i}$ and containing W discrete data points ( j=1,…,W ), the equal-time Pearson’s cross-correlation coefficient at zero lag is explicitly defined as:

$$C_{\tau =0}\left(t_{i}\right)\left\{F_{m},F_{k}\right\}=\frac{1}{W-1}\sum_{j=1}^{W}\hat{P^{\left(1\right)}}\left(F_{m},t_{i,j}\right)\hat{P^{\left(2\right)}}\left(F_{k},t_{i,j}\right)$$

where $C_{\tau =0}\left(t_{i}\right)\left\{F_{m},F_{k}\right\}$ quantifies the degree of correlation between the frequency bands *F*_*m*_ of the first muscle and *F*_*k*_ of the second muscle at time *t*_*i*_, with values ranging from $C_{\tau =0}\left(t_{i}\right)\left\{F_{m},F_{k}\right\}$ = −1 (fully anti-correlated) to $C_{\tau =0}\left(t_{i}\right)\left\{F_{m},F_{k}\right\}$ = 1 (fully positively correlated), while $C_{\tau =0}\left(t_{i}\right)\left\{F_{m},F_{k}\right\}$ = 0 indicates the absence of linear correlation. Note that the lag used in the cross-correlation analysis was determined based on the delay at which the maximum correlation coefficient occurred for each exercise protocol. In the squat test, the highest values consistently appeared at zero lag (i.e., no delay). Therefore, we computed the cross-correlation values at zero lag (indicating no delay; *τ* = 0).

Ultimately, this process produced, for every frequency-band pair (i.e., link), a new time series of correlation values with 3-s resolution. Because each muscle pair includes 100 frequency-band combinations (i.e., links), the result of this step was 100 correlation time series per muscle pair, and a total of 600 for the entire network (we consider 6 muscle pairs). Similarly to the rationale described in the Step 2 in Section 3.4.1 of the Methods, the window length and step were selected according to the experimental protocol.

Step 8: Quantification of temporal variability in cross-correlation time series

In this final step, the cross-correlation time series obtained for each frequency-band pair (i.e., link) are used to quantify the temporal variability of inter-muscular coupling. This quantification includes both static and dynamic measures, which together allow the identification of differences in the magnitude and temporal structure of coupling variability across muscle pairs, frequency bands, and physiological states.

##### 3.4.2.1 Distribution profiles of cross-correlation inter-muscular values

For each pair of EMG frequency bands (i.e., link) within the six muscle pairs (LegL-LegR, LegL-BackL, LegL-BackR, LegR-BackL, LegR-BackR, BackL-BackR), we constructed histograms of cross-correlation distribution profiles. The cross-correlation values $C_{\tau =0}\left(t_{i}\right)\left\{F_{m},F_{k}\right\}$ , ranging from −1 to 1, were divided into bins of size Δc = 0.05. The histogram was then rescaled by the maximum number of counts within the distribution. The resulting distribution profiles reflect the probability density of inter-muscular coupling between different EMG frequency bands (i.e., links), capturing their short-timescale dynamics within 3-s windows.

##### 3.4.2.2 Static measures of the temporal variability of inter-muscular interactions

To quantify the magnitude and distribution of fluctuations in the temporal variability of inter-muscular interactions, we computed three statistical measures for each intermuscular cross-correlation time series for each pair of frequency band (i.e., link) within all muscle pairs (LegL-LegR, LegL-BackL, LegL-BackR, LegR-BackL, LegR-BackR, BackL-BackR): (i) SD, (ii) kurtosis, and (ii) skewness.

The SD quantifies the magnitude of fluctuations, indicating how much inter-muscular coupling strength changes around its mean. A higher SD suggests higher variability in the interactions. In contrast, lower cross-correlation values indicate more stable coupling between muscles (Schmitt et al., 2009). The kurtosis measure quantifies the spikiness of the cross-correlation distribution. A high kurtosis value indicates more frequent extreme fluctuations in inter-muscular interactions, while lower kurtosis values reflect a more homogeneous fluctuation pattern, where extreme values are less common (Prendergast et al., 2013; Quitadamo et al., 2018; Xiang et al., 2020). Finally, the skewness assesses the asymmetry of the cross-correlation distribution. A positive skew suggests that stronger inter-muscular couplings are more frequent than weaker ones, whereas a negative skew indicates a dominance of weak or negative couplings over time (Letham and Raij, 2011; Xiang et al., 2020).

##### 3.4.2.3 Dynamic measures of the temporal variability of inter-muscular interactions - detrended fluctuation analysis (DFA)

Finally, to explore the temporal structure of variability in inter-muscular coupling, we applied Detrended Fluctuation Analysis (DFA) to the inter-muscular cross-correlation time series for each pair of frequency band within all muscle pairs (LegL-LegR, LegL-BackL, LegL-BackR, LegR-BackL, LegR-BackR, BackL-BackR).

We used the DFA method (Peng et al., 1994), which has been developed to quantify fractal correlations embedded in nonstationary signals, to estimate dynamic scale-invariant characteristics in cross-correlation fluctuations. The performance and limitations of the DFA method have been systematically studied in relation to signal features typically encountered in physiological data recordings: (1) types of trends (Hu et al., 2001); (2) random outliers/spikes, noisy segments, signals composed of parts with different correlation (Chen et al., 2002); (3) nonlinear filters (Chen et al., 2005); (4) missing data (Ma et al., 2010); (5) signal coarse-graining procedures (Xu et al., 2011) and comparing DFA performance with moving average techniques (Xu et al., 2005). Specifically, for a cross-correlation time series $C\left(t_{i}\right)$ of total length N with mean $\bar{C}$ , we first compute the integrated time series $Y\left(k\right)=\sum_{i=1}^{k}\left[C\left(t_{i}\right)-\bar{C}\right]$ . The DFA method then quantifies the root-mean-square of the detrended fluctuations, explicitly defined as:

$$F\left(n\right)=\sqrt{\frac{1}{N}\sum_{k=1}^{N}{\left[Y\left(k\right)-Y_{n}\left(k\right)\right]}^{2}}$$

where $Y_{n}\left(k\right)$ represents the local trend in each window of timescale n . A power-law functional form $F\left(n\right)\;∼\;n^{\alpha }$ indicates the presence of self-similar organization in the fluctuations. The parameter α , called the scaling exponent, quantifies the correlation properties of the signal: if α=0.5 , there is no correlation and the signal is white noise; if α=1.5 , the signal is a random walk (Brownian motion); if 0.5 <α <1.5 , there are positive correlations, where large cross-correlations are more likely to be followed by large correlations (and the same is true for small cross-correlations); if α<0.5 the signal is anticorrelated.

Further, we used the magnitude and sign approach (Ashkenazy et al., 2001; 2003) decomposing the cross-correlation variability time series into magnitude and sign of the consecutive increments in coupling strength, explicitly defined as $\Delta C\left(t_{i}\right)=C\left(t_{i+1}\right)-C\left(t_{i}\right)$ . This variability time series was decomposed into a magnitude series ( $\left|\Delta C\left(t_{i}\right)\right|$ ) and sign series ( $\text{sgn}\left[\Delta C\left(t_{i}\right)\right]$ ), which were shown to carry information about the nonlinear and linear characteristics respectively of the cross-correlation time series. These magnitude and sign characteristics were related to the intrinsic mechanisms of physiological systems control (Kantelhardt et al., 2002; Karasik et al., 2002; Hu et al., 2004; Schmitt and Ivanov, 2007; Ivanov et al., 2009).

### 3.5 Cardio-muscular coupling and network interactions

#### 3.5.1 Heart rate time series

Heartbeat detection in EKG signals relies on the identification of R waves, which occur in a quasi-periodic manner (Christov, 2004). To extract R-wave peaks, we applied the Pan-Tompkins algorithm (Pan and Tompkins, 1985), a widely used methods for detecting QRS complexes. This algorithm consists of several sequential steps, including bandpass filtering, differentiation, squaring of samples, smoothing with a moving average filter, correlation analysis and thresholding (Sieciński et al., 2020).

To compute the instantaneous HR, we took the inverse of the resulting R–R intervals and multiply the result by 60 to obtain beats per minute (BPM). Next, we applied cubic spline interpolation to generate a new HR time series with a 1-s resolution, ensuring alignment with the EMG frequency bands’ time series.

To minimize the impact of artifacts in HR recordings, we implemented a correction procedure. At each data point, if the HR value exceeded or fell below two SDs of the average HR computed within a 10-s window, it was replaced with the mean HR value within that specific window.

#### 3.5.2 Global measures of cardio-muscular coupling

The steps to quantify global measures of cardio-muscular coupling, we followed the same steps described for inter-muscular coupling (see Section 3.4), with the difference that EMG frequency-band time series are cross-correlated with the HR time series instead of another EMG frequency band time series.

Steps 1 and 2: Spectral decomposition of EMG signals and EMG frequency bands time series

The spectral decomposition of EMG signals followed the same methodology described in Section 3.4.1.

Step 3: Cross-correlations between time series of heart rate and EMG frequency bands

For each heart–muscle pair (Heart-LegL, Heart-LegR, Heart-BackL, and Heart-BackR), we computed the bivariate equal-time Pearson’s cross-correlation between heart rate (HR) and all EMG spectral power frequency bands *F*_*m*_, where *m* = 1, … ,10. This led to 1 × 10 = 10 cross-correlation values *C* for each heart–muscle pair. The value of *C* quantifies the degree of coupling between HR and the EMG frequency band *F*_*m*_ from one muscle. The cross-correlation values range from *C* = −1 (indicating full anti-correlation) to *C* = 1 (indicating full positive correlation), with *C* = 0 indicating no linear relationship between the HR and the power time series of a given EMG frequency band. As in the inter-muscular coupling (see Section 3.4.1), the time lag was selected based on the delay that yielded the highest correlation coefficient for the exercise protocol. In the squat test, the maximum correlations *C* consistently occurred at zero lag (i.e., no delay; *τ* = 0). Therefore, all heart–muscle cross-correlation values were computed at zero lag.

Step 4: Cardio-muscular cross-correlation matrices

Group-averaged cardio-muscular cross-correlation matrices summarize the coupling degree between HR and the ten EMG frequency bands for each heart-muscle pair. Each matrix consists of 1 × 10 elements, where each value represents the group-averaged cross-correlation coefficient between HR and a specific EMG frequency band.

Step 5: Network maps of cardio-muscular coupling

A multiplex network was constructed to visualize interactions between all heart-muscle pairs (i.e., sub-networks) and their hierarchical organization within the network. The graphical approach we utilized was essential for identifying universal patterns in the cardio-muscular network and understanding the hierarchical organization of sub-networks and modules. Each muscle was represented by a semicircle, with color-coded nodes representing distinct frequency bands *F*_*m*_. The network links corresponded to the cross-correlation values *C* obtained from the cross-correlation analysis and reflected the coupling strength between HR and a given frequency band from the muscle (i.e., link) within the sub-network (heart-muscle pair). The strength of these links was indicated by line color and width, providing a visual representation of the strength of interactions. In the examples used here we used: (1) weak links (0 < *C* < 0.10; very thin grey lines), (2) intermediate links (0.10 < *C* < 0.20; thin green lines), (3) strong links (0.20 < *C* < 0.30; thick dark blue lines), and (4) very strong links (*C* > 0.30; very thick magenta lines). Link-strength classification should be determined by the researcher according to the obtained results to facilitate visualization.

##### 3.5.2.1 Additional control analyses for global measures of cardio-muscular coupling

As described for inter-muscular coupling, to assess the physiological relevance of cardio-muscular coupling it is recommended to (i) conduct a Fourier phase randomization surrogate test on the recorded EMG signals (see Section 3.4.1), (ii) compute a physiological relevance threshold, and (iii) assess the effects of potential trends embedded in the HR and EMG spectral power time series.

#### 3.5.3 Temporal variability within states and evolution across states in cardio-muscular coupling

As in inter-muscular coupling (see Section 3.4.2), to investigate how cardio-muscular coupling evolves over time, we assessed the temporal variability of coupling between HR and EMG frequency bands.

Step 6: Splitting heart rate and EMG frequency band time series into time windows

We divided the HR and EMG frequency bands time series obtained in Step 2 into *NL* segments, each of length 6-s with a 3-s step (*NL* = (*N*/*L*)), where *N* is the length of the time series. In this step, both the HR and EMG frequency band time series were normalized separately within each 6-s segment to have zero mean (*μ* = 0) and unit SD (*σ* = 1). Note that the length of the time window can and should be adjusted to the type of movement and protocol used in the study.

Step 7: Cross-correlations between heart rate and EMG frequency band time series

For each heart–muscle pair (Heart-LegL, Heart-LegR, Heart-BackL and Heart-BackR) and for each 6-s segment, we calculated the bivariate equal-time Pearson’s cross-correlation between HR and all 10 EMG frequency bands (*F*_*m*_, where *m* = 1, … ,10) within each 6-s segment. This led to 1 × 10 = 10 cross-correlation values *C* (i.e., links) per heart-muscle pair per segment. Each correlation coefficient computed at time *t*_*i*_ (the midpoint of the *i*th segment) and zero lag was denoted as:

$$C_{\tau =0}\left(t_{i}\right)\{F_{m}\left.,\text{HR}\right\}$$

where $C_{\tau =0}\left(t_{i}\right)\{F_{m}\left.,\text{HR}\right\}$ quantifies the degree of correlation between the power time series of a given EMG frequency band *F*_*m*_ and HR at time *t*_*i*_, with values ranging from $C_{\tau =0}\left(t_{i}\right)\{F_{m}\left.,\text{HR}\right\}$ = −1 (fully anti-correlated) to $C_{\tau =0}\left(t_{i}\right)\left\{F_{m},\text{HR}\right\}$ = 1 (fully positively correlated), while $C_{\tau =0}\left(t_{i}\right)\left\{F_{m},\text{HR}\right\}$ = 0 indicates the absence of linear correlation. As described in the inter-muscular coupling (see Section 3.4.2.), the time lag for cross-correlation was selected based on the delay yielding the maximum correlation coefficient *C* for the given exercise protocol. In the squat test, the highest values consistently occurred at zero lag (indicating no delay; τ = 0). Therefore, cross-correlation values were computed at zero lag.

Ultimately, this process produced, for every HR and EMG frequency-band pair (i.e., link), a new time series of correlation values with 3-s resolution. Because each heart-muscle pair includes 10 HR-frequency-band combinations (i.e., links), the result of this step was 10 new correlation time series per heart-muscle pair, and a total of 40 for the entire network (we consider 4 muscles). Similarly to the rationale described in Section 3.4.1 of the Methods, the window length and step will be selected according to the experimental protocol.

Step 8: Quantification of temporal variability in cross-correlation time series

In line with inter-muscular coupling, the cross-correlation time series obtained for each heart–muscle frequency-band pair (i.e., link) are used to quantify the temporal variability of cardio-muscular coupling. This quantification includes both static and dynamic measures, which together allow the identification of differences in the magnitude and temporal structure of coupling variability across heart–muscle pairs, links, and physiological states (e.g., Rest, Exercise).

##### 3.5.3.1 Distribution profiles of cross-correlation cardio-muscular values

For each pair of HR and EMG frequency bands (i.e., link) within each heart-muscle pair (Heart-LegL, Heart-LegR, Heart-BackL, Heart-BackR), we constructed a histogram of the cross-correlation distribution by dividing the range [−1,1] into *Δc* = 0.05. Each histogram was then rescaled by the maximum number of counts, resulting in a distribution profile proportional to the probability density of cardio-muscular coupling between HR and EMG frequency bands at short timescales of 3-s. See methodological details in Section 3.4.2.

##### 3.5.3.2 Static measures of the temporal variability of cardio-muscular interactions

To quantify the magnitude and distribution of fluctuations in the temporal variability of cardio-muscular cross-correlation time series, we obtained the SD, kurtosis and skewness of each time series for each pair of HR and frequency band (i.e., link) within all heart-muscle pairs (Heart-LegL, Heart-LegR, Heart-BackL, Heart-BackR). For further details, see Section 3.4.2.

##### 3.5.3.3 Dynamic measures of the temporal variability of cardio-muscular interactions - detrended fluctuation analysis

To assess the temporal structure of variability in cardio-muscular coupling, we applied DFA to the cardio-muscular cross-correlation time series for pair of HR and frequency band (i.e., link) within all heart-muscle pairs (Heart-LegL, Heart-LegR, Heart-BackL, Heart-BackR). For a detailed description of the procedure, see Section 3.4.2.

### 3.6 Respiratory-muscular coupling and network interactions

#### 3.6.1 Breathing rate time series

To extract the breathing rate (BR) time series, several methods can be used. One option is a peak detector approach, as applied in cardio-muscular coupling analysis (see Section 3.5). However, this method is only reliable when signals are relatively free of noisy and movement artifacts, such as resting recordings. For exercise-related recordings, a time-frequency representation (TFR) of the respiratory waveform is more appropriate. TFR is a powerful tool for extracting instantaneous frequencies from different types of physiological signals, enabling the combination of time- and frequency-domain analyses (see Iatsenko et al., 2016 for details; the corresponding Matlab codes, as well as other useful time–frequency analysis tools, are freely available in https://github.com/luphysics/MODA). Since TFR requires time windows to obtain spectral power, selecting a suitable window size was necessary. We used a 20-s window (which includes at least four respiratory cycles) with 0.5-s step, producing a BR time series with 0.5-s resolution. Next, we applied a 2-s moving average filter to smooth the BR time series.

#### 3.6.2 Global measures of respiratory-muscular coupling

The analysis of the global measures of respiratory-muscular coupling (Steps 1–5) was obtained following the same procedure described for cardio-muscular coupling (see Section 3.5.2), with one modification: instead of using the HR time series, we used BR time series. All analytical steps—including cross-correlations between time series of BR and EMG frequency bands, and construction of respiratory-muscular networks—were performed following the same procedures, with the exception of the windowing parameters (i.e., window length and step), as detailed below.

Step 1 and 2: Spectral decomposition of EMG signals and EMG frequency bands time series

EMG signals were segmented into 20-s windows with a 0.5-s step, in contrast with the shorter windows used for inter-muscular (see Section 3.4.1) and cardio-muscular (see Section 3.5.1) analyses. This longer window length is essential because respiration is a very low-frequency signal, and windows of at least 20-s are required to encompass multiple respiratory cycles and to extract meaningful cross-frequency coupling components. Using shorter windows would not capture sufficient respiratory information, leading to unstable or physiologically invalid estimates. The final 0.5-s resolution of the EMG frequency band time series matches the 0.5-s resolution of the RR. All subsequent steps of the spectral decomposition and normalization followed the same procedures detailed in Section 3.4.1.

#### 3.6.3 Temporal variability within states and evolution across states in respiratory-muscular coupling

The analysis of temporal variability in respiratory–muscular coupling (Steps 6–8) followed the same procedure described for cardio-muscular coupling (see Section 3.5.3), however, using the BR time series.

## 4 Results

The following sections illustrate the successive steps involved in applying the ACFC method to quantify multisystem coordination, using body-weight squats as an example. These expected results are provided as illustrative examples of the outcome that the researcher may obtain when applying the ACFC method to empirical data. Specifically, they demonstrate how ACFC generates the three network-based outcomes: inter-muscular, cardio-muscular, and respiratory-muscular coupling.

### 4.1 Global measures

Steps 1-3: Time series of EMG spectral power frequency bands, heart rate, and breathing rate

**FIGURE 4:**
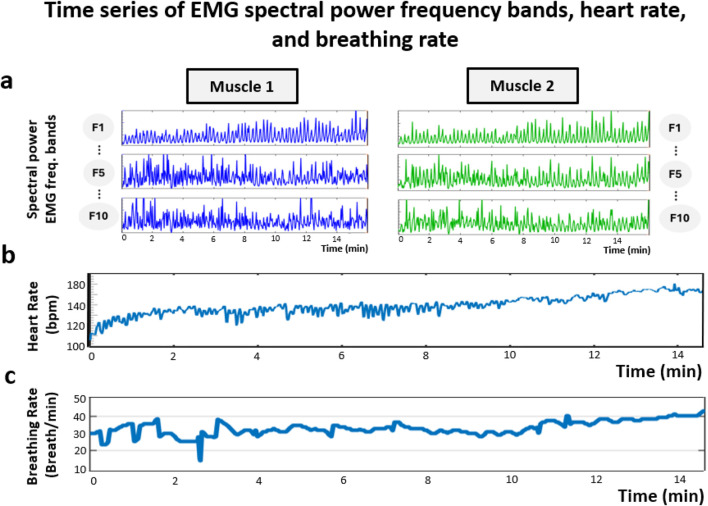
Time series of EMG spectral power frequency bands, heart rate (HR), and breathing rate (BR). **(a)** Representative EMG spectral power time series for three specific frequency bands (F1, F5, and F10) obtained from two muscles (Muscle 1 and Muscle 2) during squat exercise. **(b)** Heart rate (HR) and **(c)** Breathing rate (BR) during the same squat exercise bout as in **(a)**. EMG frequency-band time series exhibit continuous modulation throughout the exercise, whereas HR and BR show a characteristic gradual increase and a reduction in variability as fatigue accumulates. The time resolution of the instantaneous signals differs across modalities: instantaneous HR was computed at 1-s resolution, instantaneous BR at 0.5-s resolution, while instantaneous muscle spectral power was estimated at 1-s resolution for inter-muscular and cardio-muscular analyses, and at 0.5-s resolution for respiratory-muscular analyses. Note that the EMG, HR, and BR time series shown in this figure were obtained from different participants and are presented solely as illustrative examples of each signal.

From the raw EMG, EKG, and respiratory signals, time series of the spectral power are obtained for each EMG frequency band (F1 … F10) across all recorded muscles, together with HR and BR (see Sections 3.4.1, 3.5.1, 3.6.1). As illustrated in Figure 4, EMG frequency-band time series exhibit continuous modulation throughout the exercise, whereas HR and BR show a characteristic gradual increase accompanied by a reduction in variability as fatigue accumulates. These simultaneous time series constitute the inputs for all subsequent analyses.

Step 4: Cross-correlation matrices and bar charts.

**FIGURE 5:**
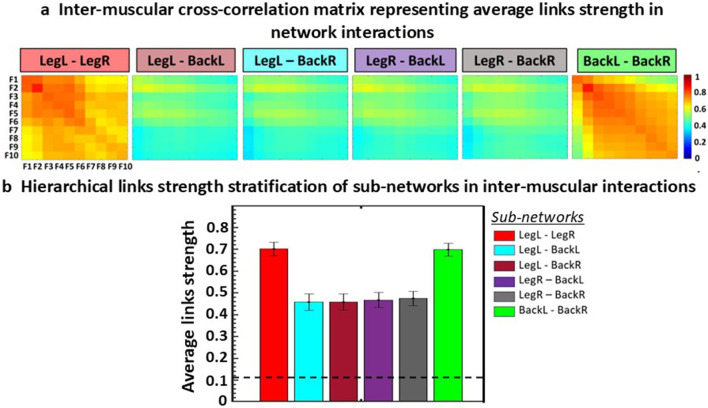
Inter-muscular cross-correlation matrices and hierarchical links strength stratification of sub-networks in inter-muscular interactions. **(a)** Representative inter-muscular cross-correlation matrices showing the coupling between spectral power across the ten EMG frequency bands for each muscle pair. In each matrix, rows correspond to the frequency bands *F*_*m*_ of the first muscle and columns to the frequency bands *F*_*k*_ of the second muscle. Color intensity indicates the strength of the cross-correlation *C(F*_*m*_ *, F*_*k*_ *)*, reflecting the degree of coupling between frequency bands across muscles. In this example, same-type muscle pairs (LegL–LegR, BackL–BackR) generally exhibit stronger link strength compared with different-type muscle pairs (LegL–BackL, LegL–BackR, LegR–BackL, and LegR–BackR). **(b)** Bar plots summarizing the global cross-correlation values for each muscle pair, obtained from matrices in **(a)**. Each bar represents the average links strength across all frequency-band combinations for a given muscle pair. Error bars represent the standard error, and the black dotted line represents the physiological significance threshold Th=0.11 (Section 3.4.1). Inter-muscular coupling is consistently lower for different-type muscle pairs than for same-type muscle pairs. These examples in the figure illustrate the procedure for inter-muscular coupling, the same steps apply to cardio-muscular and respiratory-muscular coupling.

Cross-correlation matrices are computed to quantify coupling strength between frequency bands across muscles (inter-muscular), between HR and EMG frequency bands (cardio-muscular), and between BR and EMG frequency bands (respiratory-muscular). Figure 5a presents representative inter-muscular cross-correlation matrices illustrating the correlation between the ten EMG frequency bands across all muscle pairs. Each matrix corresponds to a specific muscle pair and summarizes the interactions between frequency bands of the two muscles.

In this example, same-type muscle pairs (LegL-LegR, BackL-BackR) typically exhibit stronger links strength compared with different-type muscle pairs (LegL-BackL, LegL- BackR, LegR-BackL, and LegR-BackR). To facilitate comparison and interpretation, global coupling is summarized using bar charts (Figure 5b), where each bar represents the average links strength for a given muscle pair. As illustrated in Figure 5b, inter-muscular coupling is typically lower for different-type muscle pairs than for same-type muscle pairs.

Although this example focuses on inter-muscular coordination, the same procedure applies to cardio-muscular and respiratory-muscular coordination. The only difference lies in the dimensionality of the cross-correlation matrices. While inter-muscular coupling yields 10 × 10 matrices, cardio-muscular and respiratory-muscular coupling produce 1 × 10 matrices, reflecting coupling between HR or BR and the ten EMG spectral power frequency bands (see Sections 3.4.1, 3.5.2, 3.6.2).

Step 5: Network representation.

To visualize interactions among muscles, the heart, and respiration, as well as their hierarchical organization, multiplex networks composed of multiple sub-networks are constructed from the cross-correlation matrices.

**FIGURE 6:**
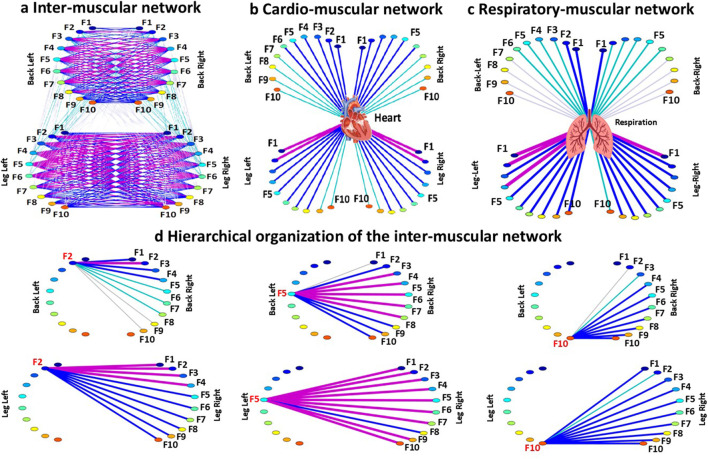
Network representations of multisystem coordination during exercise. **(a)** Inter-muscular network illustrating the coupling between EMG frequency bands across muscles. This network is generated from the cross-correlation matrices, where each link represents the coupling strength between frequency bands of different muscles. Each muscle is shown as semicircle with color nodes representing the different frequency bands (F1 … F10) within a muscle. The line width and color of the connecting lines illustrate the average links strength between frequency bands, with thicker/darker lines indicating stronger interactions. In this example, stronger coupling is observed for same-type muscle pairs (LegL–LegR, BackL–BackR), whereas different-type muscle pairs (LegL–BackL, LegL–BackR, LegR–BackL, and LegR–BackR) exhibit weaker coupling. **(b)** Cardio-muscular network showing the coupling between heart rate (HR) and EMG spectral power across muscles. Muscles are represented as semicircles with nodes for frequency bands (F1-F10). Links depict the coupling strength between HR and each EMG frequency bands within each muscle. Stronger coupling is observed between HR and lower EMG frequency bands [F1–F5]. A similar hierarchical structure is observed for Heart–Leg and Heart–Back pairs, although coupling strength is slightly reduced for Heart–Back pairs. **(c)** Respiratory-muscular network illustrating the coupling between breathing rate (BR) and EMG spectral power. For each respiratory-muscle pair, ten links quantify the coupling between BR and the ten EMG frequency bands (F1-F10). All muscles are displayed as semicircles with color-coded nodes for their frequency bands. In this example, Respiration–Leg pairs show a relatively uniform distribution of coupling strength across frequency bands, whereas Respiration–Back pairs exhibit stronger coupling predominantly in lower-frequency bands. **(d)** Hierarchical structure of links strength for the same-type muscle LegL-LegR and BackL-BackR sub-networks during exercise. Shown are only the sub-network modules for F2, F5 and F10 frequency bands extracted from the cross-correlation analyses.

Figure 6 shows representative examples of inter-muscular, cardio-muscular, and respiratory-muscular networks. In these networks, nodes correspond to EMG frequency bands, muscles are represented as semicircles, and links encode coupling strength through variations in line width and color. For the inter-muscular network (Figure 6a), link strength is classified as follows: weak links (0.05 < *C*_*m,k*_ < 0.15; very thin grey lines), intermediate links (0.15 < *C*_*m,k*_ < 0.25; thin green lines), strong links (0.25 < *C*_*m,k*_ < 0.35; dark blue thick lines) and very strong links (*C*_*m,k*_ > 0.35; magenta very thick lines). For the cardio-muscular network (Figure 6b), links strength is classified as: weak links (0 < C < 0.10; very thin grey lines), intermediate links (0.10 < C < 0.20; thin green lines), strong links (0.20 < C < 0.30; thick dark blue lines), and very strong links (C > 0.30; very thick magenta lines). For the respiratory-muscular network (Figure 6c), links strength is classified as: weak links (0.15 < C < 0.3; very thin grey lines), intermediate links (0.3 < C < 0.45; thin green lines), strong links (0.45 < C < 0.6; dark blue thick lines) and very strong links (C > 0.6; magenta very thick lines).

In the inter-muscular network shown in Figure 6a, stronger links strength is observed for same-type muscle pairs (LegL-LegR, BackL-BackR), whereas different-type muscle pairs (LegL-BackL, LegL- BackR, LegR-BackL, and LegR-BackR) exhibit weaker links strength. The cardio-muscular network (Figure 6b) illustrates a hierarchical organization characterized by stronger coupling between HR and lower EMG frequency bands [F1 … F5]. A similar hierarchical structure is observed for Heart-Leg and Heart-Back pairs, although strength is slightly reduced for Heart-Back pairs. The respiratory-muscular network (Figure 6c) shows a more uniform distribution of links strength across frequency bands for Respiration-Leg pairs, whereas Respiration-Back pairs present higher links strength predominantly in lower frequency bands.

### 4.2 Temporal variability

Steps 6 and 7: Cross-correlation time series.

To assess the temporal variability of multisystem coordination across muscles, the heart, and respiration, cross-correlation time series are generated to capture synchronous amplitude modulations at short timescales of a few seconds. These cross-correlation time series describe how links strength between EMG frequency bands, HR, and BR fluctuates over time within a given segment (e.g., Rest, Exercise).

**FIGURE 7:**
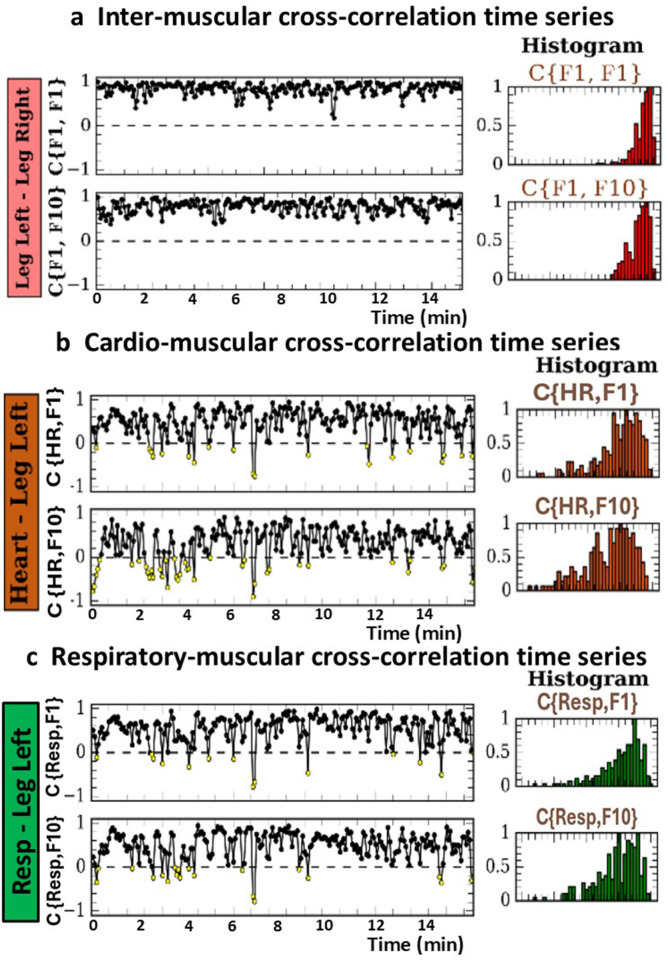
Cross-correlation time series and their respective histograms for inter-muscular, cardio-muscular, and respiratory-muscular coupling during exercise. **(a)** Inter-muscular cross-correlation time series obtained using 6-s time windows with 3-s step and zero lag (*τ* = 0). Left panels show examples of cross-correlation time series *C(t*_*i*_ *)* between pairs of EMG frequency bands time series (positive values in black, negative values in yellow). Right panels display the corresponding distribution histograms, rescaled to the peak. In this example, inter-muscular coupling variability during exercise is characterized by predominantly positive cross-correlations, relatively low variability, and narrow distributions for two representative links. **(b)** Cardio-muscular cross-correlation time series obtained using 6-s time windows with 3-s step and zero lag (*τ* = 0). Left panels show the variability in coupling between instantaneous heart rate (HR) and EMG frequency bands time series, with the associated histograms shown on the right. For two representative links, cardio-muscular coupling during exercise shows stable positive coupling with low variability and narrow histogram profiles. **(c)** Respiratory-muscular cross-correlation time series obtained using 6-s time windows with 3-s step and zero lag (*τ* = 0). Left panels show the variability in coupling between instantaneous breathing rate (BR) and EMG frequency bands time series, with the corresponding histograms on the right. These time series exhibit continuous fluctuations in coupling strength between BR and EMG frequency bands, reflecting dynamic respiratory–muscular coupling during exercise. The variability in coupling strength for different systems and subnetworks conforms to distributions with distinct functional forms.

Figure 7 presents representative examples of inter-muscular, cardio-muscular, and respiratory-muscular cross-correlation time series together with their corresponding histograms. The cross-correlation time series illustrate continuous fluctuations in coupling strength, with transitions between higher and lower correlation values. Such fluctuations reflect the dynamic reorganization of interactions among physiological systems and highlight the nonlinear and non-stationary nature of multisystem coordination. The corresponding histograms summarize the distribution of cross-correlation values across the entire segment, providing a representation of the magnitude and dispersion of coupling variability for each link.

As illustrative examples, inter-muscular coupling variability (Figure 7a) typically shows predominantly positive correlations with relatively low variability and narrow distributions. Cardio-muscular coupling variability (Figure 7b) similarly exhibits stable positive coupling with low variability. Respiratory-muscular coupling variability (Figure 7c) shows continuous fluctuations in coupling strength between BR and EMG frequency bands, with distributional profiles comparable to those observed for cardio-muscular coupling.

Step 8: Quantification of temporal variability in cross-correlation time series

In this final step, the cross-correlation time series obtained for each link are used to quantify temporal variability in multisystem coordination. This quantification includes both static measures, which summarize the magnitude and distribution profiles of coupling fluctuations within a segment, and dynamic measures, which characterize the temporal organization and structure of these fluctuations across timescales, as described in Sections 3.4.2, 3.5.3, 3.6.3.

## 5 Discussion

The aim of this methodological article was to provide a detailed, step-by-step description of the analytical procedures used by ACFC to quantify multisystem coordination across muscles, the heart, and respiration, addressing the lack of a unified framework for cross-frequency, multisystem coupling analysis during dynamic exercise. ACFC uses simultaneous EMG, EKG, and respiratory signals as inputs to quantify pairwise coupling between muscle-muscle, heart-muscle, and respiration-muscle systems. The analyses ultimately generate three network-based markers: inter-muscular, cardio-muscular, and respiratory-muscular coupling. By combining global measures of coupling within a defined segment (e.g., Rest, Exercise) with analyses of temporal variability that capture how coordination evolves over time, ACFC provides a comprehensive characterization of multisystem coordination.

One of the major strengths of the ACFC method is its flexibility, which enables researchers to adapt the method to a wide range of movements, exercise protocols, and specific objectives. At the parameter level, several components of the analysis can be customized, such as the number and width of EMG frequency bands, the window size and step used to generate spectral power time series, and the selection of muscles for EMG acquisition. At the system level, researchers can tailor the analysis by selecting specific muscles for EMG acquisition and by choosing whether to compute one or all three network-based markers (inter-muscular, cardio-muscular, or respiratory-muscular coordination), depending on the research question. At the analysis level, the method can be applied either to global measures of coupling within predefined segments or extended to include analyses of temporal variability that capture how coordination evolves over time.

Given the complexity of the ACFC outcome, several practical recommendations should be considered when applying the method. First, we suggest focusing on a single network-based marker, as presenting multiple markers simultaneously may reduce interpretability. Second, for inter-muscular measures, it is recommendable to include no more than four muscles in the analysis to avoid excessive network dimensionality. Third, figures illustrating the experimental protocol should always be included, as they facilitate both understanding of subsequent analyses and reproducibility of the study. Additionally, providing representative examples of spectral power time series and HR or BR time series helps contextualize group-averaged cross-correlation values. Finally, we recommend presenting cross-correlation matrices together with bar charts summarizing all correlations, as well as global network visualizations to capture the hierarchical organization of interactions across systems.

While this manuscript focuses on muscular, cardiac, and respiratory data, the ACFC method can be extended to other high-frequency physiological signals, provided that they are not substantially affected by movement-related artifacts during exercise (e.g., accelerometry, near-infrared spectroscopy, cardiac impedance). Although electroencephalography (EEG) signals have been used to investigate cortico–muscular coupling using related approaches (Rizzo et al., 2020; 2023), EEG recordings are particularly susceptible to motion artifacts during dynamic exercise, which currently limits their suitability for ACFC analyses in this context. Future developments in wearable sensor technology and artifact-reduction methods may, however, enable the extension of ACFC to additional signal modalities under control or low-movement conditions.

## 6 Limitations and future perspectives

Some limitations of the current implementation of ACFC should be acknowledged. At present, the method is effective and sensitive for cyclic movements with a stable tempo, as the method relies on windowed spectral decomposition that requires regular cycles. Furthermore, the method can only be reliably applied to signals acquired at high sampling frequencies (≥500 Hz), which may limit its use with datasets or devices that record at lower temporal resolution.

A relevant practical consideration is that EKG-derived rate measures and belt-based respiratory signals capture important aspects of cardiovascular and ventilatory regulation, but they do not fully represent more functionally proximal outputs such as stroke volume, cardiac output, or gas exchange. Therefore, coupling patterns involving these variables should be interpreted primarily as reflecting coordination at the level of regulatory responses, rather than as direct coupling between primary functional outputs. Future developments of the ACFC method may benefit from incorporating more functionally proximal variables, such as beat-to-beat stroke volume estimates, non-invasive cardiac output surrogates, flow-based ventilation measures, capnography, or VO_2_/VCO_2_, while recognizing the practical and technical challenges associated with the simultaneous acquisition of these signals during movement-based exercise protocols.

Regarding Phase 2 of the ACFC method (quantifying temporal variability in physiologic coupling), a limitation is that Pearson correlations are computed within relatively short time windows, which may contain a limited number of samples and therefore produce noisier cross-correlation estimates. Future methodological work may further refine statistical validation strategies for these short-window coupling estimates to confirm that the observed temporal variability in systems interactions exceeds sampling noise.

In addition, several parameters within the ACFC workflow—such as window length, moving step, and frequency-band selection—may vary depending on the characteristics of the experimental protocol and the physiological signals that are analyzed. Although the present work does not include a systematic sensitivity analysis of these parameters, previous applications of the ACFC framework have produced consistent patterns of physiological network interactions across multiple individuals, datasets, and experimental conditions, including different movements (e.g., squats and push-ups; Garcia-Retortillo et al., 2023; Garcia-Retortillo and Ivanov, 2022), participant populations (young and older adults; Garcia-Retortillo and Ivanov, 2025; Garcia-Retortillo et al., 2025b), as well as male and female cohorts (Abenza et al., 2025). These observations suggest that the ACFC method is reasonably robust across experimental contexts, although future studies should further explore the sensitivity of specific parameter choices.

Finally, although the ACFC method is motivated by the complex and nonlinear nature of physiological interactions, the coupling metric used here is the Pearson cross-correlation coefficient, which captures linear relationships between the amplitude dynamics of two signals. This choice was made because Pearson correlation provides a robust and interpretable measure of synchronous co-modulation between physiological signals. Importantly, the use of a linear metric does not imply that the underlying physiological interactions are linear; rather, it provides a practical and transparent way to quantify coordinated regulatory responses between systems as represented by synchronous modulation in the spectral amplitude of the oscillatory components embedded in the output signals.

## 7 Conclusion

In summary, this methodological article provides a complete and reproducible framework for applying the ACFC method to assess multisystem coordination across muscles, the heart, and respiration. By detailing each step–from signal acquisition and spectral decomposition to the construction of inter-muscular, cardio-muscular, and respiratory-muscular networks of dynamic interactions–this work enables researchers to implement ACFC across different movements, protocols, and scientific objectives. As such, ACFC represents a valuable tool for advancing the study of coordinated physiological dynamics in exercise as well as in other physiological states and conditions, with potential relevance for broader physiological and clinical applications.

## Data Availability

The original contributions presented in the study are included in the article/supplementary material, further inquiries can be directed to the corresponding author.
